# Optimizing multi-class intrusion detection in IIoT: advanced feature selection using grey wolf optimizer and machine learning classifiers

**DOI:** 10.3389/frai.2026.1932015

**Published:** 2026-10-05

**Authors:** Ibrahim Al-Shibly, Llorenç Burgas, Joaquim Massana

**Affiliations:** Universitat de Girona - Control Engineering and Intelligent Systems, Campus Montilivi, EPS IV, Girona, Catalonia, Spain

**Keywords:** adaptive feature ranking, cybersecurity, feature selection, grey wolf optimizer, industrial internet of things, intrusion detection system, machine learning, multi-class classification

## Abstract

**Introduction:**

The rapid expansion of the Industrial Internet of Things (IIoT) has increased the cyberattack surface of industrial infrastructures, creating demand for intrusion detection systems (IDS) that combine predictive performance with deployment efficiency.

**Methods:**

This study proposes a Grey Wolf Optimizer (GWO)-guided fixed top-k feature-ranking framework for multi-class IIoT intrusion detection using candidate-dependent redundancy regularization, class-weighted learning, stratified cross-validation, repeated runs, and independent holdout evaluation. The framework was evaluated on the CIC IIoT Dataset 2025 and independently assessed on Edge-IIoTset as a secondary benchmark across six machine learning classifiers and several feature-selection baselines.

**Results:**

On CIC IIoT Dataset 2025, Random Forest and Decision Tree achieved holdout Macro-F1 scores of 0.9389 and 0.9330, respectively, using only 10 selected features, representing an 85.92% reduction from the 71-feature model-input space. Repeated training-only comparisons showed significant overall differences among GWO, BA, LightGBM, and Boruta (Friedman *χ*² = 16.920, *p* = 0.000734; Kendall’s *W* = 0.564); GWO outperformed BA and LightGBM after Holm correction but did not differ significantly from Boruta. Ablation analysis further showed lower feature redundancy under adaptive regularization, although its predictive advantage was not statistically significant. Results on Edge-IIoTset showed that feature-selection effectiveness is dataset dependent.

**Discussion:**

Overall, the proposed framework provides a practical balance between predictive performance, feature compactness, and computational efficiency for resource-constrained IIoT intrusion detection.

## 1 Introduction

The IIoT platform is a key component of modern industrial infrastructures. It enables seamless connectivity among industrial sensors, controllers, edge devices, and cloud-based analytics platforms. Such connectivity enhances industrial automation and operational intelligence. However, it also creates a broader and more accessible attack surface that can be exploited by cyber attackers (Orman, 2025).

Effective intrusion detection in Industrial IoT is difficult due to the diversity of IIoT devices and the dimensions of network-traffic feature sets, as well as the time-sensitive nature of industrial operations. Therefore, in addition to providing reliable predictive performance, IDS models need to be computationally efficient enough to run on constrained resources. Feature selection can help reduce model-complexity and increase interpretability and support real-world use cases where deployments occur at the edge in industry (Kaushik et al., 2025). New research shows that a fusion of feature selection and machine learning can lead to improved intrusion detection results; however, the problem of the trade-off between performance and efficiency remains unresolved (Ismail et al., 2025).

Recent intrusion-detection research has increasingly explored explainable deep learning, federated learning, graph neural networks, and large-language-model-assisted frameworks to improve representation learning, privacy, adaptability, and attack interpretation (Ahmad et al., 2025; Bai et al., 2025; Ma et al., 2025; Yang et al., 2025c). These developments broaden the capabilities of modern IDSs, but their computational and architectural requirements do not eliminate the need for compact feature representations suitable for resource-constrained industrial deployment.

Several methodological gaps nevertheless remain unresolved. The majority of algorithms that have been proposed for performing feature selection via optimization usually focus on the problem of optimization of classification accuracy while neglecting the aspect of having deployment-specific needs regarding low dimensionality (Maseno and Wang, 2024). Consequently, even though the algorithms yield optimized feature sets, these sets still end up having redundancies, thereby contributing to high levels of complexity of the models. Also, since many of the existing studies revolve only around binary anomaly detection, class imbalance becomes an additional problem, making it difficult to accurately evaluate performance (Musthafa et al., 2024). Furthermore, performance established on a single benchmark may not remain stable when class distributions, feature redundancy, and attack complexity change across IIoT traffic environments. In addition, under a fixed-cardinality top-k search, a penalty based only on subset size is constant across candidate solutions and therefore cannot distinguish competing subsets.

Feature selection is one of the ways in which interpretability can be improved, computational costs can be decreased and models’ generalizability can be enhanced. While filter-based feature selection approaches have high efficiency and scalability, their approach to feature selection is based on relevance criteria which makes these approaches dependent on statistical measures of dependency between variables, and does not account for feature dependencies (Li et al., 2024). Meanwhile, in wrapper-based methods feature subset evaluation is done using classification accuracy and, although being computationally costly, they are more likely to capture feature dependencies (Maseno and Wang, 2024).

To address these challenges, this paper develops a GWO-guided top-k feature-ranking framework for multi-class IIoT intrusion detection. The methodological contribution does not lie in introducing Decision Tree or Random Forest classifiers, which are established algorithms. Rather, these classifiers are deliberately used as computationally efficient and interpretable learners for evaluating whether a compact GWO-selected representation can preserve competitive multi-class detection performance while reducing deployment-related computational burden. The proposed framework formulates feature ranking under predefined low-dimensional top-k constraints and evaluates the resulting representations using class-imbalance-aware learning, stratified cross-validation, repeated optimization runs, an independent holdout set, and deployment-oriented efficiency measures. Edge-IIoTset is additionally used as a secondary benchmark; it is not presented as cross-dataset transfer validation.

The major contributions of this study are as follows:

- A GWO-guided fixed top-k feature-ranking framework for compact multi-class IIoT intrusion detection.
- A candidate-dependent adaptive redundancy-control mechanism that remains meaningful when every candidate subset has the same cardinality k .
- A leakage-safe validation protocol in which the train/test split precedes all data-dependent preprocessing, feature selection, sensitivity analysis, and model-development decisions.
- A deployment-oriented evaluation across six classifier families using Macro-F1, MCC, feature reduction, training time, inference time, and feature-selection cost.
- Repeated-run non-parametric statistical testing, sensitivity analysis for k, ablation of the redundancy term, and separate secondary-benchmark evaluation on Edge-IIoTset.

The structure of the paper is presented as follows. Section 2 reviews the state of the art in IIoT intrusion detection and feature selection. Section 3 describes the proposed methodology and optimization framework. Section 4 presents the datasets and experimental setup. Section 5 reports the results, secondary-benchmark analysis, discussion, implications, and limitations. Finally, Section 6 presents the conclusions.

## 2 State of the art

The gradual progression of traditional signature-based approaches toward intelligent machine learning systems that can cope with high dimensional industrial traffic is observed in the most recent research in modern IIoT intrusion detection studies. Initially, researchers paid attention to achieving maximum possible accuracy levels in detection through the use of advanced statistical preprocessing methods in combination with machine learning approaches (Benrhennou and Kalloubi, 2025). In operational technology scenarios, Benrhennou and Kalloubi showed that a filter-based approach toward feature selection could provide satisfactory results with reduced complexities of models and training efforts (Benrhennou and Kalloubi, 2025). At the same time, survey analyses identified scalability problems with large-scale IIoT applications, which emphasized the necessity to develop lightweight IDSs (Alakhras, 2025).

As more complexities were added to the process of analyzing IIoT traffic, wrapper-based approaches were applied more frequently in attempts to enhance feature subset selection. Shtayat et al. presented a feature selection approach based on swarm intelligence used in the process of identifying different feature subsets (Shtayat et al., 2024). Similarly, Verma et al. utilized the equilibrium optimizer to improve performance by decreasing the number of classification errors in industrial traffic using ensemble learning approaches (Verma et al., 2025). Lastly, Karim et al. proposed a framework that incorporates Bayesian machine learning and Sparrow Search Algorithm (Karim et al., 2024). However, most optimization objectives primarily focused on maximizing classification performance without explicitly incorporating deployment-oriented low-dimensional feature ranking strategies.

Recent optimization research also emphasizes that competing objectives should be formulated explicitly. Yang et al. (2025a) enhanced NSGA-II for multi-objective optimization, Yang et al. (2024b) introduced diversity enhancement into a multi-objective marine predator algorithm, and Yang et al. (2025b) integrated predictive modeling with multi-objective optimization. More broadly, Xu et al. (2026) describe AI for Science as an increasingly adaptive and closed-loop paradigm for scientific modeling and optimization, providing additional context for AI-assisted search strategies. Although these studies concern control rather than intrusion detection, they support the use of candidate-specific adaptive criteria instead of terms that remain constant across solutions.

This distinction is particularly important for fixed-cardinality feature selection. When every candidate contains exactly k features, a penalty based only on $\frac{|S|}{D}$ is identical across candidate subsets and therefore cannot discriminate between them. Accordingly, the present study uses candidate-dependent feature redundancy as the regularization quantity, allowing the optimization process to distinguish equally sized subsets according to the amount of redundant information they contain.

Alongside optimization-based works, deep learning models have gained attention for automatic extraction of hierarchical representation from complicated network traffic data. Alamro et al. (2025) proposed a deep learning approach along with advanced feature selection that showed good results in terms of intrusion detection in IoT-enabled systems. Self-attention deep convolutional neural networks were proposed by Alshehri et al. (2024) for intrusion detection in IIoT networks where the attention mechanism assigned adaptive weights to discriminatory features in traffic data. Also, explainable AI-based feature selection methods have been proposed for making deep intrusion detection models more interpretable while retaining their good classification performance. Tserenkhuu et al. (2025) used explainable AI-based feature ranking along with domain constrained features to create interpretable detection results.

Recent research has extended this trend beyond conventional deep architectures. Ahmad et al. (2025) proposed XGRU-IDS, combining Extra Trees-based feature selection, GRU-based sequential modeling, and SHAP explanations. Bai et al. (2025) examined federated intrusion-detection training for privacy-preserving distributed learning, while Ma et al. (2025) combined redundancy-aware feature selection with LLM fine-tuning. GNN-based IDS research likewise models relationships among devices or network flows rather than treating observations only as independent feature vectors (Yang et al., 2025c).

Another direction that some studies have taken concerns the feasibility and efficiency aspects of implementation. Tiwari et al. (2024) proposed an energy-efficient IDS, tailored for IIoT at the edge, where inference time is reduced through model quantization while maintaining high detection accuracy. The use of hybrid ensemble learning strategies to improve intrusion detection robustness has also received considerable attention. For example, Abou Elasaad et al. (2025) proposed AegisGuard, a multi-stage hybrid intrusion detection system that integrates optimized feature selection with a calibrated ensemble learning strategy. Shu and Lu (2025) developed a two-step botnet detection approach for IIoT that incorporates feature selection with gradient boosting algorithms, while Balhareth and Ilyas (2024) suggested tree-based classifiers together with filter-based feature importance for efficient intrusion detection in IoMT.

These works highlight dominant trends including statistical filtering for scalability, metaheuristic wrapper optimization, deeper or hybrid networks, distributed learning, and emerging graph- or LLM-assisted architectures. However, many existing architectures still prioritize prediction performance more strongly than low-dimensional feature extraction required for resource-constrained industrial use.

These developments indicate that the contemporary IDS landscape includes explainable deep learning, federated optimization, graph-based representation learning, and LLM-assisted modeling. The present study addresses a narrower and complementary question: whether a compact low-dimensional traffic representation obtained through wrapper-based optimization can retain competitive multi-class performance while reducing deployment burden. Because recent GNN-, federated-, and LLM-based IDS studies employ different datasets, label spaces, preprocessing protocols, and computational settings, their reported scores are not treated as directly comparable experimental baselines. Table 1 therefore provides a methodological SOTA comparison, whereas the controlled experiments use feature-selection baselines that can be evaluated under the identical training and holdout protocol.

**Table 1:** Comparative analysis of selected recent IIoT intrusion-detection studies.

Study	Dataset	Architecture	FS/representation	Temp.	Int.	Edge	Limitation/relevance
Benrhennou and Kalloubi (2025)	OT traffic	ML	Filter-based	No	Partial	Yes	No multi-class evaluation; scalable filtering
Maseno and Wang (2024)	IoT dataset	ELM	GA wrapper	No	Low	No	Binary focus; wrapper optimization
Shtayat et al. (2024)	IIoT traffic	ML	IBSWO	No	Low	Limited	High search cost; swarm-based selection
Verma et al. (2025)	UNSW-NB15	Ensemble	Equilibrium optimization	No	Low	No	Anomaly-oriented ensemble optimization
Karim et al. (2024)	TON_IoT	ML	Bayesian + SSA	No	Low	Partial	Limited deployment regularization
Yang et al. (2024a)	IIoT dataset	BiGRU + CNN + attention	Embedded	Yes	Low	No	Heavy deep architecture
Alamro et al. (2025)	IoT cloud	Deep network	Hybrid FS	Yes	Low	No	High resource demand
Alshehri et al. (2024)	InSDN	CNN + attention	Embedded	Yes	Limited	No	Resource intensive
Tiwari et al. (2024)	Edge-IIoTset	ML	Statistical FS	No	Partial	Yes	Lightweight edge-oriented IDS
Ahmad et al. (2025)	CICIoT2023	GRU + SHAP	Extra Trees + XAI	Yes	High	Limited	Interpretability with deep sequential modeling
Bai et al. (2025)	IoT IDS benchmarks	Federated ML/DL	Distributed learning	Varies	Partial	Yes	Privacy-preserving distributed IDS
Ma et al. (2025)	Five IoT datasets	LLM + LightGBM	mRMR + evolutionary FS	Partial	Partial	Limited	Redundancy + imbalance + LLM augmentation
Yang et al. (2025c)	IIoT traffic	GNN	Graph representation	Yes	Low	Limited	Relational modeling; greater architectural complexity
This work	CIC-IIoT-2025 + Edge-IIoTset	Six conventional classifier families	Adaptive redundancy-regularized GWO top-k	No	Partial	Deployment-oriented	Compact representation, leakage-safe holdout, statistical and cost analysis

Reference Study, Benchmark Data Set, Learning Architecture, and Feature Selection Method are specified for each work. Filter algorithms have the advantage of being highly scalable and edge-applicable, but the interaction between features is limited. Metaheuristic algorithms using wrapper methods can be useful in improving prediction quality through feature subset selection; however, many works only concentrate on classification results. Deep learning models have been found to be effective in capturing spatiotemporal correlations, but large computational cost limits their applicability in edge environments (Alamro et al., 2025). The proposed framework therefore does not attempt to compete with GNNs or LLMs in representation complexity; instead, it investigates how much of the original traffic representation can be removed while preserving competitive multi-class discrimination and quantifying computational savings.

## 3 Methodology

Figure 1 shows the overall flow chart of the proposed Intrusion Detection system architecture. The proposed intrusion detection system architecture involves three phases: Data preprocessing, adaptive GWO-guided top-k feature ranking, and multi-class classification. The above-mentioned phases convert the raw IIoT traffic data into the discriminating feature set for multi-class intrusion detection.

**Figure 1:**
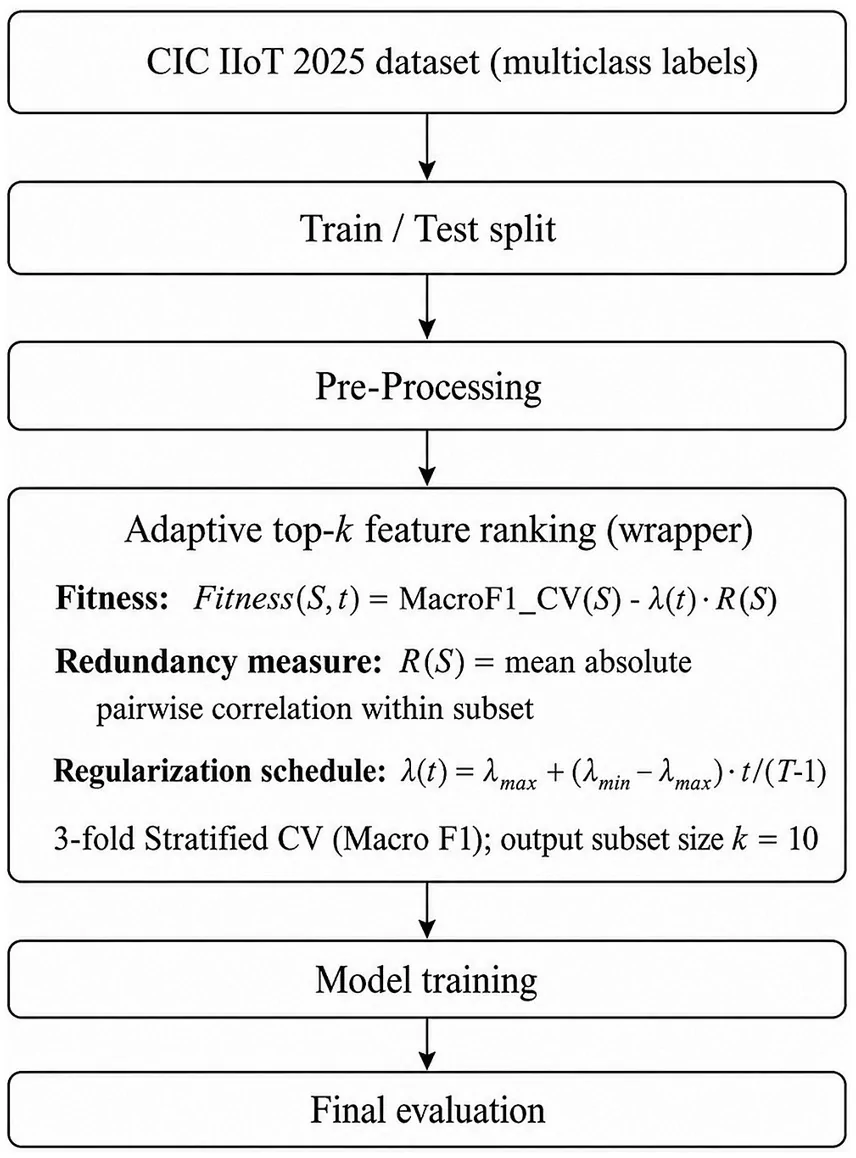
Overview of the leakage-safe GWO-guided top-k feature-ranking and evaluation workflow.

To prevent information leakage, the dataset is first divided into training and independent holdout test sets, after which all data-dependent preprocessing parameters, feature-ranking decisions, sensitivity analysis, and model-development procedures are estimated exclusively from the training data. The holdout test set remains untouched until final evaluation.

### 3.1 Data preprocessing

A strict preprocessing pipeline is applied to the CIC IIoT Dataset 2025 to ensure data quality and improve the reliability of subsequent learning stages:

1. *Train-Test Partitioning*: A predefined stratified 80/20 split was first applied to preserve the distribution of all eight category-level classes, providing 548,536 observations for model development and 137,135 observations for independent holdout evaluation. The holdout partition was not used for preprocessing estimation, feature-subset selection, selection of k , sensitivity analysis, regularization assessment, hyperparameter decisions, or optimization.
2. *Data Cleaning and Formatting*: After partitioning, identifier and timestamp variables were removed. Features with more than 40% missing values in the training partition were also excluded, and the same feature-removal decisions were then applied unchanged to the holdout partition. Attack and benign labels were mapped to the eight category-level classes used in this study.
3. *Imputation and Normalization*: Missing and infinite values were handled using median imputation, followed by Z-score standardization. Median values, feature means, standard deviations, low-variance criteria, and other data-dependent preprocessing parameters were estimated exclusively from the training partition and then applied without re-estimation to the holdout partition.

The same preprocessing principles were also applied to the secondary benchmark dataset (Edge-IIoTset), with dataset-specific identifier and leakage-prone attributes removed as appropriate. Edge-IIoTset is used as a secondary-benchmark robustness assessment rather than cross-dataset transfer validation because feature rankings and fitted model parameters are not transferred between datasets.

### 3.2 Adaptive GWO-guided top-k feature ranking

A Grey Wolf Optimizer (GWO) is used to formulate feature ranking as a continuous optimization problem. Unlike traditional optimization-based feature selection approaches that primarily maximize classification accuracy, the proposed framework introduces an adaptive regularization term within a fixed-size top-k evaluation setting to support discriminative low-dimensional feature representations suitable for IIoT deployment environments. Almomani (2020) presented a wrapper-based intrusion-detection feature-selection model that compared GWO with other swarm optimizers. In the proposed framework, GWO is used within a fixed-size top-k feature-ranking setting. Because every candidate subset contains the same number k of features, subset cardinality itself cannot distinguish competing wolves; therefore, the regularization term is defined using candidate-dependent feature redundancy.

#### 3.2.1 Optimization formulation

Each candidate solution in the proposed GWO-based framework, referred to as a wolf, is represented as a continuous weight vector $W\in {\mathbb{R}}^{D}$ , where D denotes the total dimensionality of the feature space. During optimization, a fixed top-k subset is formed by selecting the features associated with the largest weights in W for evaluation. Because ∣S∣ is identical for all candidate wolves within a given experiment, a penalty proportional to $\frac{|S|}{D}$ would be constant and would therefore have no effect on their relative fitness ranking. The fitness function instead combines cross-validated predictive performance with the average absolute pairwise correlation among the selected features.

$$\text{Fitness}(S,t)=\text{Macro}F1_{\mathrm{CV}}(S)-\lambda (t)\;R(S)$$

$$R(S)=\frac{2}{k(k-1)}\sum_{\begin{array}{c} i<j\\ i,j\in S \end{array}}|\rho_{\mathrm{ij}}|$$

$\text{MacroF}1_{\mathrm{CV}}(S)\;$ represents the mean Macro-F1 score obtained using stratified 3-fold cross-validation on the training set. R(S) represents the mean absolute pairwise correlation among the k selected features calculated from the training data only, and λ(t) represents the adaptive regularization coefficient. The first term rewards subsets that provide balanced multi-class discrimination, whereas the second discourages redundant subsets containing strongly correlated predictors. Thus, two candidate wolves containing the same number of features can receive different fitness values.

#### 3.2.2 Adaptive regularization strategy

The regularization coefficient decreases linearly during optimization:

$$\lambda (t)=\lambda_{\max}+(\lambda_{\min}-\lambda_{\max})\frac{t}{T-1};\text{with}\;\lambda_{\max}=0.05\;\text{and}\;\lambda_{\min}=0.005$$

This adaptive schedule is applied within the objective formulation during optimization under the adopted fixed top-k setting. The schedule decreases from $\lambda_{\max}=0.05$ to $\lambda_{\min}=0.005$ , giving greater weight to redundancy reduction early in the search and relatively greater emphasis to Macro-F1 later. Its contribution is evaluated against no regularization and fixed λ=0.01 .

#### 3.2.3 Algorithm description

Only the modifications required for the proposed feature-ranking framework are summarized here; the standard GWO hunting and position-update equations follow Mirjalili et al. (2014). This avoids repeating the conventional description of GWO while keeping the proposed changes explicit.

**ALGORITHM 1**

Adaptive redundancy-regularized GWO top-k feature ranking

◦ Initialize *P* wolf weight vectors and identify *α*, *β*, and *δ* wolves.
◦ For each iteration t, compute *λ*(*t*).
◦ For each wolf, select the k features with the largest weights.
◦ Evaluate the subset by stratified 3-fold Macro-F1 using a class-weighted Decision Tree surrogate.
◦ Compute *R*(*S*) from training data and calculate $\;\text{Fitness}(S,t)=\text{MacroF}1_{\mathrm{CV}}(S)-\lambda (t)R(S)$ .
◦ Update *α*, *β*, *δ* and the remaining wolf positions using the standard GWO equations.
◦ Return the top-k subset associated with the final *α* wolf.

#### 3.2.4 Comparative feature-selection baselines

The proposed framework was compared with feature-selection approaches representing different methodological families: Bat Algorithm (BA) as a population-based metaheuristic wrapper, LightGBM feature importance as an embedded ranking approach, Boruta as an all-relevant tree-based method, and the complete feature space without feature selection.

The no-feature-selection configuration provides a reference for assessing the performance–dimensionality trade-off, whereas Boruta provides a less aggressively compressed all-relevant representation, BA enables comparison with another bio-inspired optimizer, and LightGBM provides an embedded tree-based ranking baseline. Thus, the comparison evaluates GWO against heterogeneous feature-selection mechanisms rather than only GWO variants.

#### 3.2.5 Ablation and sensitivity design

To isolate the contribution of redundancy regularization from the underlying GWO search process, three configurations were evaluated under identical population size, iteration count, k , surrogate classifier, cross-validation folds, and random seeds:

1 GWO without regularization:

$$\text{Fitness}(S)=\text{MacroF}1_{\mathrm{CV}}(S)$$

2 GWO with fixed redundancy regularization:

$$\text{Fitness}(S)=\text{MacroF}1_{\mathrm{CV}}(S)-0.01R(S)$$

3 Proposed adaptive GWO:

$$\text{Fitness}(S,t)=\text{MacroF}1_{\mathrm{CV}}(S)-\lambda (t)R(S)$$

where λ(t) decreases from 0.05 to 0.005 during optimization.

This ablation separates the contribution of the GWO search itself from that of redundancy penalization and the additional effect of adapting the regularization strength over iterations.

A sensitivity analysis was additionally performed for k∈{6,7,10} using the same training-only cross-validation protocol to determine whether the final k=10 setting was supported by the data rather than selected arbitrarily.

### 3.3 Multi-class classification framework

After feature ranking, the dataset is projected onto the selected low-dimensional feature space. Unlike binary anomaly detection approaches, the proposed framework performs multi-class intrusion classification to identify specific attack categories. The Decision Tree classifier is used as the primary surrogate learner during optimization due to its low inference latency, computational efficiency, and interpretability in real-time IIoT environments. During wrapper optimization, the Decision Tree surrogate uses class-balanced weighting to reduce majority-class dominance. For downstream evaluation, class weighting is applied where supported by the classifier, while Macro-F1 provides a common imbalance-aware endpoint across all six classifier families.

For final evaluation, six classifier families are considered: Random Forest (Breiman, 2001), Decision Tree (Breiman et al., 1984), Logistic Regression (Cox, 1958), Gaussian Naive Bayes (John and Langley, 1995), HistGradientBoosting (Friedman, 2001), and Multi-Layer Perceptron (Rumelhart et al., 1986). These established classifiers are used to assess whether the selected representation remains useful across different inductive biases rather than to claim classifier novelty. Final evaluation and statistical analysis follow the protocol described in Section 3.4.

### 3.4 Evaluation strategy and statistical analysis

An independent 20% holdout partition was reserved for final evaluation and remained inaccessible throughout preprocessing fitting, feature optimization, selection of k , sensitivity analysis, ablation analysis, and classifier development. Within the training partition, stratified three-fold cross-validation was used for feature-subset evaluation and model-selection decisions. Once a configuration was selected, the corresponding classifier was retrained using the complete training partition and evaluated once on the untouched holdout partition.

Performance was evaluated using Accuracy, macro-averaged Precision, macro-averaged Recall, Macro-F1, and the multiclass Matthews Correlation Coefficient (MCC). Macro-F1 was treated as the primary predictive measure because each class contributes equally regardless of prevalence, while MCC provides a complementary measure that remains informative under class imbalance.

Deployment efficiency was quantified using feature-selection time (fs_time_s), classifier training time (train_time_s), and total holdout inference time (inference_time_ms). Feature reduction was reported as both the number of retained features and the percentage reduction relative to the dataset-specific post-preprocessing feature space, calculated as:

$$\text{Feature Reduction}(\%)=(1-\frac{k}{D})\times 100,$$

where k is the number of retained features and D is the number of available model-input features. Thus, k=10 corresponds to an 85.92% reduction from 71 features for CIC IIoT Dataset 2025 and a 58.33% reduction from 24 features for Edge-IIoTset.

Because all candidate subsets have the same cardinality k , dimensional compactness is controlled explicitly through k , whereas adaptive regularization acts on candidate-specific redundancy R(S) . The two mechanisms therefore serve distinct purposes: k controls representation dimensionality, while R(S) guides the optimizer toward less redundant subsets of the prescribed size. Sensitivity analysis was conducted for k∈{6,7,10} using training-only cross-validation, and the final k=10 setting was selected before the holdout set was accessed.

The main comparative experiments were repeated using the same 10 random seeds for all competing feature-selection methods, allowing the results to be paired by experimental run. Non-parametric tests were selected because normality of the repeated-run performance differences was not assumed. Macro-F1 differences were first assessed using the Friedman omnibus test. When significant, two-sided Wilcoxon signed-rank tests compared GWO with each competing method, with Holm correction applied for multiple comparisons. Kendall’s W was reported as the omnibus effect-size measure and rank-biserial correlation as the pairwise effect-size measure. Statistical significance was assessed at α=0.05 .

The Edge-IIoTset analysis followed the same dataset-specific development and holdout protocol. Because preprocessing parameters, feature rankings, and classifier models were fitted independently for Edge-IIoTset rather than transferred from CIC IIoT Dataset 2025, this experiment is described as a secondary-benchmark robustness assessment rather than external cross-dataset transfer validation.

## 4 Materials and experimental setup

This section presents the datasets, parameter settings, evaluation metrics, and experimental configuration used to assess the proposed IIoT intrusion detection framework. Particular attention is given to leakage prevention, separation of model development from holdout evaluation, feature-subset sensitivity, repeated-run evaluation, statistical testing, and deployment-oriented computational cost.

### 4.1 Dataset

This study used the publicly accessible CIC IIoT Dataset 2025. This is a real-time sensor-based benchmark for testing intrusion detection systems in industrial IoT networks. It has 685,671 entries of network traffic with 94 attribute types that describe each entry. In total, there are 284,999 attack entries (41.56%) and 400,672 benign entries (58.44%). As stated by Firouzi et al. (2025), the dataset includes 7 broad categories of attacks and 50 detailed attack scenarios. Examples include, but are not limited to, TCP SYN Flood, UDP Flood, and HTTP Flood.

The definition of the classification process in this study has been set at the level of categories. Thus, in terms of the final experimental setup for CIC IIoT Dataset 2025, there were 8 classes consisting of 7 attack categories plus one benign class. In such an experimental setup, there is still imbalance within the classes in relation to the data. For instance, reconnaissance traffic constitutes 15.44% of all the data, DoS attacks constitute 8.42%, and DDoS attacks make up 8.27%. On the other hand, brute force attacks make up 0.88% of all the data. The audited class counts were benign 400,672; reconnaissance 105,848; DoS 57,736; DDoS 56,692; MITM 25,490; malware 24,177; web attacks 9,040; and brute force 6,016. This imbalance motivated the use of stratified data partitioning, class-weighted learning, and Macro-F1 as the principal optimization criterion.

Although the dataset dimensionality is moderate, the feature space exhibits redundancy and multicollinearity among several traffic attributes. Consequently, low-dimensional feature ranking becomes important for reducing unnecessary complexity while preserving discriminative information required for reliable multi-class intrusion detection. After deterministic removal of identifiers and leakage-safe preparation of numerical predictors, 71 numerical model-input features remain. The stratified 80/20 split contains 548,536 training observations and 137,135 untouched holdout observations; therefore, k=10 corresponds to an 85.92% feature reduction.

A second benchmark, Edge-IIoTset, was used to assess whether the behavior observed on CIC IIoT Dataset 2025 remains consistent in a different IIoT traffic environment. Edge-IIoTset (Ferrag et al., 2022) is a realistic cyber-security benchmark designed for IoT and IIoT intrusion detection in edge-enabled environments. The original dataset contains 2,219,201 traffic instances described using 61 extracted features and was generated from a heterogeneous seven-layer testbed involving cloud, fog, edge, SDN, blockchain, NFV, and IoT/IIoT perception components. It includes benign traffic together with 14 attack types grouped into five major threat categories. After dataset-specific preprocessing and removal of identifier and leakage-prone attributes, 24 numerical model-input features remained; therefore, retaining 10 features corresponds to a 58.33% reduction on Edge-IIoTset. The Edge-IIoTset experiment is treated as a secondary-benchmark robustness assessment rather than cross-dataset transfer validation; no model parameters or selected feature subsets learned from CIC IIoT Dataset 2025 are directly transferred to Edge-IIoTset.

### 4.2 Experimental setup and parameter settings

The dataset was preprocessed according to the methodology described previously. Non-numeric identifier columns were removed, missing values were imputed using median imputation, and Z-score normalization was applied to standardize all features. To prevent information leakage, the stratified 80/20 train-test partition was established before estimating data-dependent preprocessing parameters, which were fitted exclusively on the training partition and then applied unchanged to the holdout partition. The proposed GWO-guided top-k feature-ranking framework uses a population size of 10 candidate solutions and 30 optimization iterations. During optimization, a class-weighted Decision Tree classifier is used as the surrogate learner, while Macro-F1 is adopted as the primary objective within stratified 3-fold cross-validation.

For comparative evaluation, the proposed approach is compared with the Bat Algorithm (BA), LightGBM-based feature importance, Boruta, and a Without FS setting. The same paired run structure is used for the main training-only comparison, while k∈{6,7,10} and the no/fixed/adaptive regularization configurations are evaluated as training-only sensitivity and ablation analyses before the holdout set is accessed.

All feature-selection and downstream-classifier settings were fixed before the holdout set was accessed; no model or feature-selection hyperparameter was tuned using holdout performance. Exact implementation settings and random seeds are provided in the accompanying public code repository.

## 5 Results and discussion

Various experiments were performed on the CIC IIoT Dataset 2025 in order to evaluate the effectiveness of the adaptive GWO-based top-k feature ranking approach. The goal was to discover compact and discriminatory low-dimensional feature sets that enhance the multi-class IDS while keeping the deployment requirements in mind. The suggested approach was compared with benchmarking methods including biologically inspired, embedded, all-relevant, and without-feature-selection approaches: BA (Yang, 2010), LightGBM (Ke et al., 2017), Boruta (Kursa and Rudnicki, 2010), and the Without FS setting. The assessment is based on classification models representing different learning families and IIoT practitioner constraints.

The training-only comparison shows that GWO achieved the highest mean cross-validated Macro-F1 among the compact 10-feature methods, while Boruta obtained comparable predictive performance with a substantially larger subset. The sensitivity analysis supports k=10 within the tested compact range. The ablation results show that adaptive regularization produced the lowest mean redundancy, whereas predictive differences among the three regularization configurations were small. A Friedman test across the ablation conditions yielded $\chi^{2}=2.529,p=0.2823$ therefore, the adaptive term is interpreted primarily as a redundancy-control mechanism rather than as a statistically demonstrated source of higher Macro-F1.

From a predictive perspective, the strongest results for the compact GWO representation are obtained with the tree-based classifiers. Random Forest achieves holdout Macro−F1=0.9389 and Decision Tree achieves 0.9330 using only 10 of 71 input features, corresponding to an 85.92% reduction. These results indicate that compact feature ranking is particularly compatible with tree-based decision boundaries, while the lower scores for Logistic Regression and Gaussian Naive Bayes show that the selected representation remains classifier dependent.

The cross-method holdout comparison provides a better perspective on this trade-off. For Decision Tree, GWO achieves Macro−F1=0.9330 , compared with 0.9399 for BA, 0.9411 for LightGBM, 0.9552 for Boruta, and 0.9554 without feature selection. Accordingly, GWO should not be interpreted as the absolute predictive winner; its main advantage lies in retaining strong predictive performance with a much more compact 10-feature representation. For Gaussian Naive Bayes, the ordering changes substantially, which further confirms that the effect of feature selection depends on the downstream classifier.

The four-method Friedman test is significant ( $\chi^{2}=16.920,\mathrm{df}=3,p=0.000734$ ) with ${\text{Kendall}}^{’}s\;W=0.564$ . Holm-adjusted Wilcoxon tests show that GWO outperforms BA (adjusted p=0.027344 ; rank-biserial = +0.855) and LightGBM (adjusted p=0.005859 ; rank-biserial = +1.000), whereas the GWO-Boruta difference is not significant after correction (adjusted p=0.275391 ). These results support a specific interpretation rather than an overall superiority claim: GWO provides strong training-only feature-selection performance while remaining statistically comparable with Boruta under the paired runs.

Figure 2 provides an intuitive overview of training-only feature-selection behavior together with the predictive and computational behavior of the GWO top-10 representation across the six classifiers. Figure 3 complements this by showing the five-method holdout comparison for Decision Tree and Gaussian Naive Bayes. The related quantitative results are reported in Tables 2–6.

**Figure 2:**
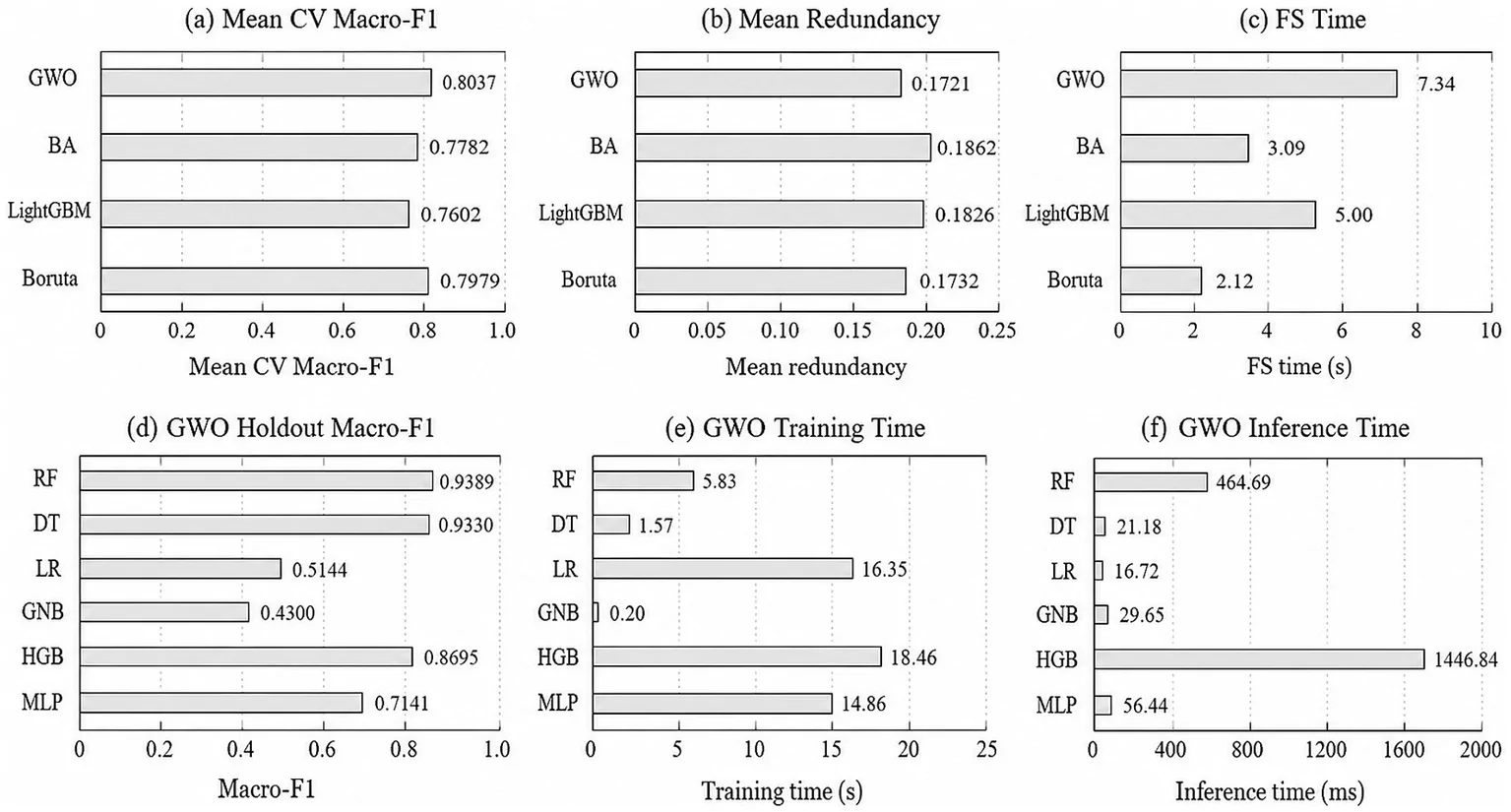
Summary of **(a)** training-only mean cross-validated Macro-F1, **(b)** mean feature redundancy, **(c)** feature-selection time, **(d)** GWO top-10 holdout Macro-F1, **(e)** GWO classifier training time, and **(f)** GWO inference time on the CIC IIoT Dataset 2025.

**Figure 3:**
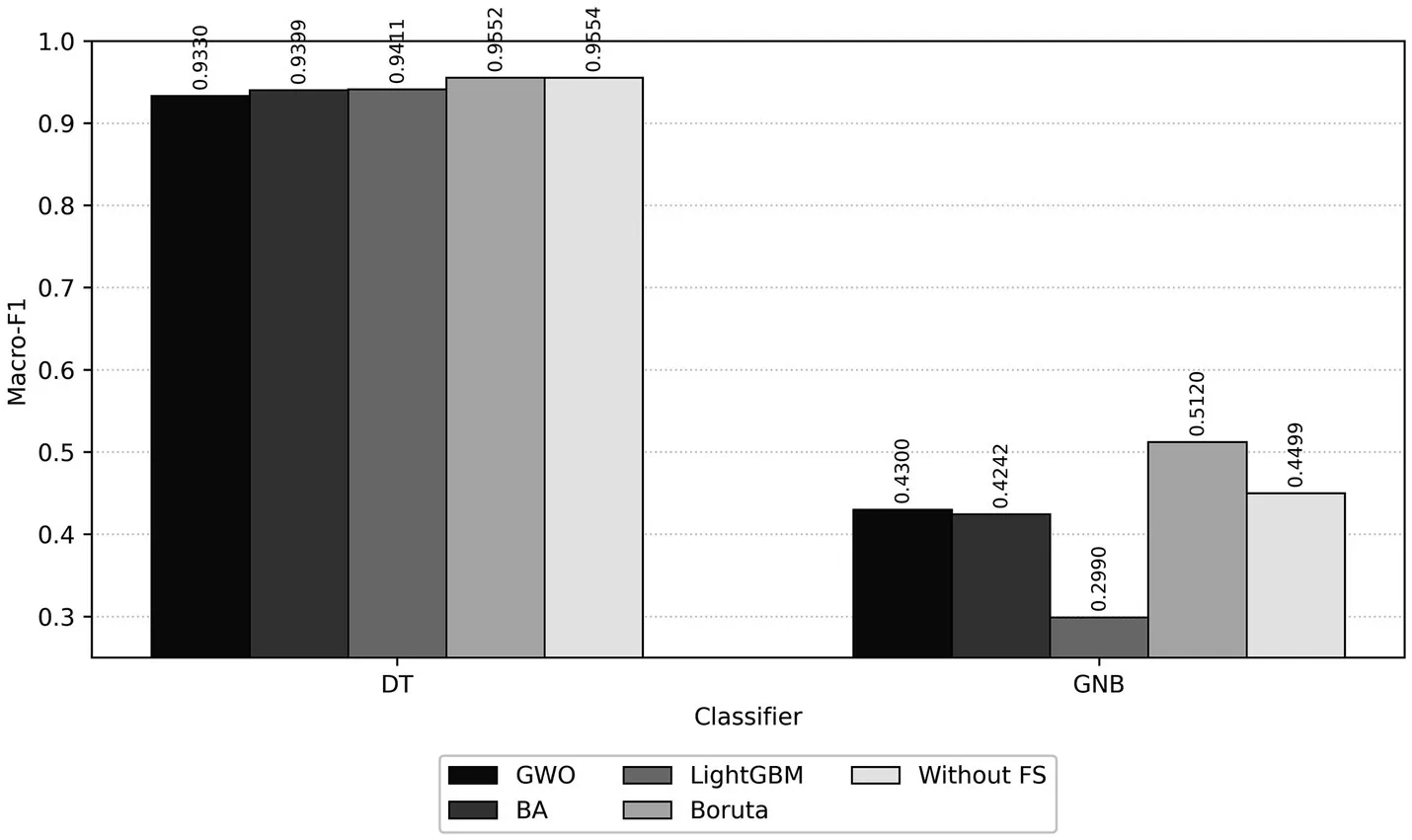
Holdout Macro-F1 comparison of GWO, BA, LightGBM, Boruta, and the complete feature space for Decision Tree and Gaussian Naive Bayes.

**Table 2:** Training-only feature-selection comparison on CIC IIoT Dataset 2025.

Method	Runs	Selected features	Reduction (%)	Mean CV Macro-F1	SD	Mean redundancy	Mean FS time (s)
GWO	10	10.0	85.92	0.8037	0.0129	0.1721	7.34
BA	10	10.0	85.92	0.7782	0.0272	0.1862	3.09
LightGBM	10	10.0	85.92	0.7602	0.0002	0.1826	5.00
Boruta	10	59.2	16.62	0.7979	0.0003	0.1732	2.12

**Table 3:** Sensitivity of k and ablation of redundancy regularization.

Analysis	Setting	Runs	Reduction (%)	Mean CV macro-F1	SD	Mean redundancy
*k* sensitivity	*k* = 6	10	91.55	0.7724	0.0227	0.1904
*k* sensitivity	*k* = 7	10	90.14	0.7860	0.0208	0.1494
*k* sensitivity	k=10	10	85.92	0.8037	0.0129	0.1721
Ablation	No regularization	9	85.92	0.8032	0.0098	0.1891
Ablation	Fixed λ=0.01	9	85.92	0.8056	0.0104	0.1818
Ablation	Adaptive λ:0.05→0.005	9	85.92	0.8055	0.0124	0.1722

Sensitivity results are based on 10 training-only runs. Ablation results are based on nine paired runs completed under identical experimental settings across the three regularization configurations. The Friedman test across the ablation configurations yielded $\chi^{2}=2.529,p=0.2823$ .

**Table 4:** Independent holdout performance of the GWO-selected top-10 CIC representation across six classifiers.

Classifier	Accuracy	Macro-Precision	Macro-Recall	Macro-F1	MCC	Features	Reduction (%)	Train time (s)	Inference time (ms)
RF	0.9600	0.9609	0.9187	0.9389	0.9349	10	85.92	5.83	464.69
DT	0.9593	0.9575	0.9108	0.9330	0.9337	10	85.92	1.57	21.18
LR	0.7338	0.5239	0.5942	0.5144	0.5905	10	85.92	16.35	16.72
GNB	0.7070	0.5180	0.4999	0.4300	0.5275	10	85.92	0.20	29.65
HGB	0.9311	0.8538	0.9009	0.8695	0.8878	10	85.92	18.46	1446.84
MLP	0.8534	0.6991	0.7817	0.7141	0.7687	10	85.92	14.86	56.44

The GWO top-10 representation was selected using training-only cross-validated Macro-F1. The independent 20% holdout partition was accessed only after feature-selection and model-development decisions were completed.

**Table 5:** Cross-method holdout comparison for Decision Tree and Gaussian Naive Bayes.

Classifier	Method	Accuracy	Macro-precision	Macro-recall	Macro-F1	MCC	Features	Reduction (%)
DT	GWO	0.9593	0.9575	0.9108	0.9330	0.9337	10	85.92
DT	BA	0.9577	0.9642	0.9178	0.9399	0.9311	10	85.92
DT	LightGBM	0.9606	0.9656	0.9189	0.9411	0.9358	10	85.92
DT	Boruta	0.9671	0.9787	0.9338	0.9552	0.9465	60	15.49
DT	Without FS	0.9672	0.9791	0.9338	0.9554	0.9467	71	0.00
GNB	GWO	0.7070	0.5180	0.4999	0.4300	0.5275	10	85.92
GNB	BA	0.7013	0.5331	0.4951	0.4242	0.5212	10	85.92
GNB	LightGBM	0.4381	0.4272	0.4016	0.2990	0.3187	10	85.92
GNB	Boruta	0.5273	0.6135	0.5851	0.5120	0.4317	60	15.49
GNB	Without FS	0.4435	0.5459	0.5660	0.4499	0.3760	71	0.00

For each feature-selection method, the final feature subset was selected using training-only cross-validation before independent holdout evaluation. No holdout result was used to select the reported subset.

**Table 6:** Repeated-run statistical comparison of training-only feature-selection Macro-F1.

Comparison	Wilcoxon *W*	Raw *p*	Holm-adjusted *p*	Rank-Biserial	GWO wins/losses
GWO vs. BA	4.0	0.013672	0.027344	+0.855	8/2
GWO vs. LightGBM	0.0	0.001953	0.005859	+1.000	10/0
GWO vs. Boruta	16.0	0.275391	0.275391	+0.418	7/3

The omnibus Friedman test across GWO, BA, LightGBM, and Boruta yielded. $\chi^{2}=16.920,p=0.000734$ ; *Kendall’s* W=0.564 . Pairwise Wilcoxon signed-rank tests were two-sided and Holm-adjusted across the three comparisons.

Generally, the findings indicate that the GWO-guided top-k feature-ranking framework provides a competitive and compact representation when combined with tree-based classifiers and offers a deployment-oriented alternative for multi-class IIoT intrusion detection. The proposed method presents a useful compromise regarding prediction efficacy, compactness of the selected subset, and computational cost. Where Boruta or the complete feature space obtains somewhat better predictive performance, GWO retains the practical advantage of a substantially smaller representation rather than predictive superiority.

### 5.1 Secondary-benchmark results on edge-IIoTset

The additional Edge-IIoTset experiment examines whether the behavior observed on CIC IIoT Dataset 2025 remains stable in a different traffic environment. Because its preprocessing, feature ranking, and classifier models are fitted independently, the experiment is interpreted as secondary-benchmark robustness rather than cross-dataset transfer (Table 7).

**Table 7:** Mean secondary-benchmark predictive performance, selected subset size, and feature reduction on Edge-IIoTset.

Classifier	Method	Accuracy	Precision	Recall	Macro-F1	MCC	Selected features	Feature reduction (%)
RF	GWO	0.8974	0.8624	0.9009	0.8648	0.8872	10.0	58.33
RF	BA	0.8655	0.8308	0.8669	0.8321	0.8523	10.0	58.33
RF	LightGBM	0.8975	0.8301	0.8607	0.8284	0.8874	10.0	58.33
RF	Boruta	0.9004	0.8816	0.9044	0.8735	0.8910	9.6	60.00
RF	Without FS	0.9052	0.8554	0.9112	0.8682	0.8957	24.0	0.00
DT	GWO	0.8846	0.8510	0.8898	0.8538	0.8730	10.0	58.33
DT	BA	0.8490	0.8147	0.8525	0.8175	0.8339	10.0	58.33
DT	LightGBM	0.8816	0.8170	0.8463	0.8145	0.8697	10.0	58.33
DT	Boruta	0.8854	0.8680	0.8911	0.8605	0.8742	9.6	60.00
DT	Without FS	0.8906	0.8420	0.8984	0.8556	0.8795	24.0	0.00
LR	GWO	0.5885	0.5573	0.6342	0.5616	0.5505	10.0	58.33
LR	BA	0.4643	0.4406	0.5197	0.4361	0.4160	10.0	58.33
LR	LightGBM	0.5764	0.5145	0.5865	0.5247	0.5357	10.0	58.33
LR	Boruta	0.5192	0.4811	0.5728	0.4769	0.4765	9.6	60.00
LR	Without FS	0.6829	0.6432	0.7204	0.6623	0.6515	24.0	0.00
GNB	GWO	0.3090	0.3119	0.3803	0.2885	0.2699	10.0	58.33
GNB	BA	0.2381	0.2749	0.3127	0.2251	0.1899	10.0	58.33
GNB	LightGBM	0.3371	0.2438	0.3790	0.2701	0.2993	10.0	58.33
GNB	Boruta	0.4096	0.3493	0.4766	0.3481	0.3695	9.6	60.00
GNB	Without FS	0.2317	0.2905	0.3146	0.2346	0.1751	24.0	0.00
HGB	GWO	0.9066	0.8941	0.8966	0.8808	0.8973	10.0	58.33
HGB	BA	0.8689	0.8601	0.8504	0.8368	0.8563	10.0	58.33
HGB	LightGBM	0.9009	0.8770	0.8604	0.8368	0.8912	10.0	58.33
HGB	Boruta	0.9065	0.9264	0.8585	0.8751	0.8971	9.6	60.00
HGB	Without FS	0.9082	0.8854	0.9102	0.8852	0.8991	24.0	0.00
MLP	GWO	0.8844	0.8913	0.8579	0.8584	0.8727	10.0	58.33
MLP	BA	0.8376	0.8455	0.8123	0.8094	0.8218	10.0	58.33
MLP	LightGBM	0.8775	0.8743	0.8241	0.8158	0.8652	10.0	58.33
MLP	Boruta	0.8864	0.9106	0.8419	0.8585	0.8750	9.6	60.00
MLP	Without FS	0.8917	0.8846	0.8837	0.8702	0.8809	24.0	0.00

Feature reduction is calculated relative to the 24-feature Edge-IIoTset model-input space. Edge-IIoTset is treated as a secondary-benchmark robustness assessment rather than cross-dataset transfer validation; preprocessing parameters, feature rankings, and classifier models are fitted independently for this dataset.

The results on Edge-IIoTset are generally lower than on CIC IIoT Dataset 2025 and show that relative feature-selection performance is dataset dependent. For Random Forest, GWO obtains Macro-F1 = 0.8648, compared with 0.8735 for Boruta and 0.8682 without feature selection. For Decision Tree, the corresponding values are 0.8538, 0.8605, and 0.8556. Thus, the predictive penalty associated with the compact GWO representation remains relatively limited for these tree-based learners, although it is no longer advantageous in predictive terms. The ranking changes further for Logistic Regression, Gaussian Naive Bayes, HistGradientBoosting, and MLP, confirming an interaction between dataset properties, the selected representation, and classifier behavior.

The data in Table 8 indicate that the reduced performance on Edge-IIoTset is related to structural differences between the datasets rather than to a single feature-selection technique. Edge-IIoTset contains 15 target classes compared with 8 classes in CIC IIoT Dataset 2025, and its class distribution is substantially more uniform, as reflected by normalized class entropy of 0.9492 compared with 0.6520 and a top-five class share of 0.4713 compared with 0.9428. CIC IIoT Dataset 2025 also contains more highly correlated feature pairs (64 compared with 11). The first 10 principal components explain 90.64% of standardized variance in CIC IIoT Dataset 2025 but only 68.39% in Edge-IIoTset. These findings show why a fixed value of k does not represent the same amount of retained information across datasets and should therefore not be assumed to be universally optimal.

**Table 8:** Structural comparison of the two benchmark feature spaces.

Dataset	Classes	Top-5 class share	Normalized class entropy	Corr. pairs ∣r∣≥0.80	Top-10 PCA variance	Final model-input features	Top-10/features
CIC IIoT Dataset 2025	8	0.9428	0.6520	64	0.9064	71	0.1408
Edge-IIoTset	15	0.4713	0.9492	11	0.6839	24	0.4167

### 5.2 Discussion, implications, and limitations

On the whole, the obtained results suggest that the developed framework based on GWO-guided top-k feature selection provides an efficient and viable compromise between prediction accuracy, compactness of selected subsets, and application-related efficiency in the case of multi-class IIoT intrusion detection. The main benefits of the method can be seen in the analysis of the CIC IIoT Dataset 2025, where compact 10-feature subsets retain strong prediction performance while simplifying the feature space. On the other hand, the additional results on Edge-IIoTset demonstrate that the efficiency of feature selection depends greatly on the traffic environment as well as on the choice of classifiers.

The experiments focus on conventional tabular classifiers and do not claim direct superiority over recent GNN-, federated-, explainable deep-learning-, or LLM-based IDS architectures, which address partly different objectives and computational regimes. These directions are therefore treated as complementary rather than direct substitutes, and future work may integrate the proposed redundancy-aware feature-ranking objective with more advanced learning architectures.

Several limitations should be considered when interpreting these findings. First, the fixed top-k design improves experimental control and deployment predictability but does not optimize subset cardinality directly, and the Edge-IIoTset findings show that a single k may not be equally appropriate for datasets with different redundancy structures. Second, the use of a Decision Tree as the wrapper surrogate may favor representations that are especially compatible with tree-based decision boundaries. Third, although two IIoT benchmarks were evaluated, the second dataset represents independent secondary-benchmark validation rather than cross-dataset transfer; no feature subset or fitted model learned from CIC IIoT Dataset 2025 was transferred directly to Edge-IIoTset. Fourth, the ablation indicates that adaptive redundancy regularization lowers within-subset redundancy but does not provide a statistically significant predictive advantage over fixed or absent regularization. Finally, the observed advantages should be interpreted jointly in terms of prediction, dimensionality, and computational cost rather than predictive accuracy alone.

## 6 Conclusion

This study addressed the challenge of developing an intrusion detection framework that simultaneously achieves high predictive performance and deployment efficiency in Industrial Internet of Things (IIoT) environments. The findings demonstrate that GWO-guided fixed top-k feature ranking supports effective multi-class intrusion detection while producing compact feature representations. By combining wrapper-based optimization, class imbalance handling, stratified cross-validation, repeated-run statistical analysis, and independent holdout evaluation within a unified framework, the proposed approach offers a systematic methodology for balancing detection performance with computational efficiency under deployment-oriented computational constraints. The objective uses candidate-dependent redundancy rather than a cardinality term that is constant across equal-sized candidates.

Unlike approaches seeking to optimize performance via ever-higher dimensional feature spaces, this paper shows that a substantially reduced feature representation can retain competitive classification performance for several classifier families. On CIC IIoT Dataset 2025, the fixed 10-feature GWO representation reduces the 71-feature model-input space by 85.92% and achieves holdout Macro-F1 scores of 0.9389 with Random Forest and 0.9330 with Decision Tree. Repeated training-only statistical testing showed significant overall differences among GWO, BA, LightGBM, and Boruta; GWO outperformed BA and LightGBM after Holm correction but did not differ significantly from Boruta. The holdout comparisons also show that Boruta or the complete feature space can obtain slightly higher predictive scores for some classifiers. Therefore, the contribution should be interpreted as a compact and competitive performance-efficiency trade-off rather than universal predictive superiority.

The main achievement of this work is to incorporate compact wrapper-based feature ranking with deployment-oriented and statistically supported evaluation within a unified framework for IIoT intrusion detection. Such an approach helps enhance existing feature selection techniques, showing that the ability to predict, feature set size, and computation cost should be treated collectively rather than in conflict with each other. The Edge-IIoTset results further reinforce that feature-selection effectiveness depends on dataset structure, class complexity, and the downstream classifier. Future work should examine adaptive feature cardinality, multi-surrogate or multi-objective optimization, direct cross-dataset transfer, and integration with federated, graph-based, and LLM-assisted IDS architectures.

## Data Availability

Publicly available datasets were analyzed in this study. This data can be found at: CIC IIoT Dataset 2025 (DataSense): Official Canadian Institute for Cybersecurity (CIC) Dataset Portal: https://www.unb.ca/cic/datasets/iiot-dataset-2025.html. No accession number available. Edge-IIoTset: IEEE DataPort repository. DOI: https://doi.org/10.21227/mbc1-1h68.
